# Does the Frequency of Watching Television Matters on Overweight and Obesity among Reproductive Age Women in Ethiopia?

**DOI:** 10.1155/2020/9173075

**Published:** 2020-08-01

**Authors:** Mohammed Ahmed, Abdu Seid, Adnan Kemal

**Affiliations:** ^1^Epidemiology, Woldia University, Woldia, Ethiopia; ^2^Maternity and Reproductive Health Nursing, Woldia University, Woldia, Ethiopia; ^3^Human Nutrition, Defense University, Addis Ababa, Ethiopia

## Abstract

**Background:**

Studies in developed countries have revealed an association of different magnitudes between watching television and the risk of being overweight and obese among reproductive age women. Even so, there is no evidence of such an association in the context of the Ethiopian population. Hence, the study aimed to assess the association between watching television with overweight and obesity in a nationally representative sample of Ethiopian women.

**Methods:**

A cross-sectional study was conducted by using secondary data analysis from 2016 Ethiopia demographic and health survey among women aged from 15 to 49 years. The samples were selected using a two-stage stratified cluster sampling technique. A total of 10,074 women were included in the analysis. The outcome variables were both overweight and obesity, whereas the main exposure variable was the frequency of watching television. Multivariate logistic regression analysis was performed for adjusting potential confounders. Adjusted odds ratio (AOR) with 95% confidence intervals was used to declare a statistically significant association.

**Results:**

The study found that watching television at least once a week was significantly associated with both overweight (AOR: 1.79; 95% CI: 1.20–2.73) and obesity (AOR: 3.76; 95% CI: 2.04–6.95). The study also divulged that the odds of overweight were higher among women aged 25–39 years (AOR: 2.17; 95% CI: 1.25–3.77) and 40–49 years (AOR: 2.69; 95% CI: 1.45–5.00), urban residents (AOR: 1.76; 95% CI:1.17–2.65), attended higher education (AOR:2.11; 95% CI: 1.22–3.65), and richest in the wealth index (AOR: 2.83; 95% CI:1.71–4.68). Similarly, the odds of obesity were higher among women aged 25–39 years and 40–49 years, attended higher education, and the richest in wealth index.

**Conclusions:**

The results from this study demonstrated that watching television at least once a week is associated with obesity among reproductive age women in Ethiopia. Therefore, a social behavioral change communication campaign needs to be taken to improve awareness regarding the harmful consequences of watching television for long hours. Further research studies should be conducted among men and adolescents to determine whether this positive association exists among that target population as well.

## 1. Background

Overweight and obesity are defined as abnormal or excessive fat accumulation that may impair health. Obesity is a worldwide public health problem, which affects both developed and developing countries [1]. Overweight and obesity are important risk factors for the development of noncommunicable diseases, including preeclampsia and eclampsia [2], cardiovascular diseases [3], hypertension [4], diabetes mellitus [5, 6], chronic kidney diseases [7], and cancer [8].

Reproductive aged women have higher rates of overweight and obesity and are more adversely affected by obesity-related complications than men [9]. This gender discrepancy is mainly owed to general weight gain during pregnancy, childbearing years, adverse lifestyle, or risk factors associated with pregnancy and the postpartum period [10]. Maternal obesity increases the risk of copious complications, including a higher risk of cesarean delivery, a higher incidence of anesthetic and postoperative complications, low Apgar scores, macrosomia, and neural tube defects [11, 12].

The association between watching television with overweight and obesity was evidenced by a different explanation such as hours spent in front of the television, decrease in the physical activity [13], a television-based food advertising influencing what and how people eat [14], and watching television leads to mindless eating or a lack of attention paid to consume due to external cues in the environment [15].

In the same vein, different studies revealed that watching television is positively associated with an overall increase in food intake [16, 17], particularly pizza [17], fast food, and high-calorie snacks [18] and is inversely associated with intakes of vegetables and fruits [19, 20]. Similarly, young adults consumed more calories from energy-dense foods when watching television than when listening to classical music [17].

Various studies in different countries showed that watching television frequently was associated with overweight and obesity among reproductive aged women [21–24]. A previous study, which was conducted in Ethiopia, on the determinant of obesity and overweight among women [25] faced two drawbacks; first, the study was conducted without excluding women who gave birth in the last 2 months, which overestimated the prevalence of overweight and obesity as evidence showed that postpartum body weight is influenced by gestational weight gain and lactation [26, 27]. Second, both overweight and obesity were analyzed as one outcome, merged during their analysis, which leads to misclassification of the outcomes. Therefore, the current study endeavored to fill this dearth by investigating whether there is an association between the frequency of watching television with overweight and obesity among reproductive age women.

## 2. Methods

### 2.1. Data Source and Sampling Procedure

The current study uses secondary data from the 2016 Ethiopia demographic and health survey (EDHS). A detailed description of the study design and methodology of the survey were founded elsewhere [28]. A two-stage stratified cluster sampling was used. Since Ethiopia has nine regional states and two city administrations, stratification was done by separating each structural division into urban and rural areas, except Addis Ababa (entirely urban). Therefore, a total of 23 sampling strata have been created. Then, each stratum was again further divided into enumeration areas (EAs) or clusters prepared by the 2007 Population and Housing Census as a sampling frame. In the first stage, a total of 645 EAs were selected. Of which, 202 were from urban areas. In the second stage, a fixed number of 28 households per cluster were selected randomly from the household listing. A total of 15,683 women (15–49 years) were interviewed, making up response rates of 95%. The analytic sample for the current study consisted of women who were not pregnant and gave birth 2 months or more with a BMI of greater or equal to 18.5 Kg/m^2^ were included in the analysis (*n* = 10074).

### 2.2. Selection Criteria

**Figure 1:**
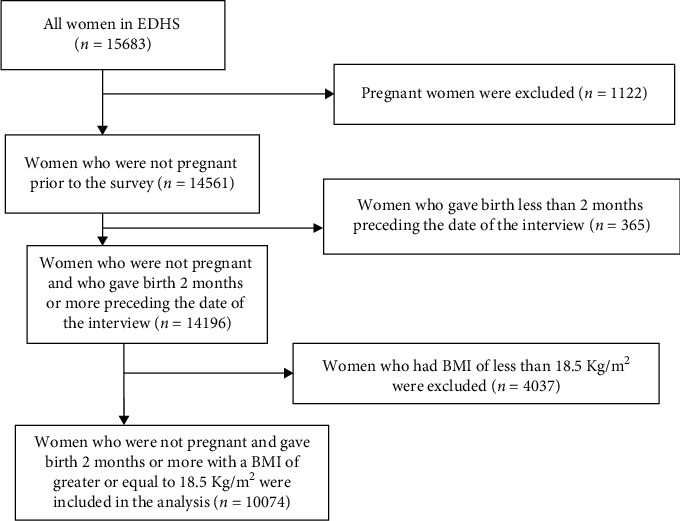
Flow chart showing the weighted sample used in the study.

The sample utilized in this study excluded (i) women who were pregnant at the time of the survey (*n* = 1122); (ii) women who gave birth less than 2 months preceding the date of the interview (*n* = 365); and (iii) women who had a BMI of less than 18.5 Kg/ m^2^ (*n* = 4037) (Figure 1).

### 2.3. Study Variables

The dependent variables of this study had two outcomes (overweight and obesity) measured by body mass index (BMI), which is expressed as weight in kilogram divided by height in m^2^. EDHS carries out anthropometric measurements such as height and weight for women aged 15–49 years, excluding women who are pregnant and women who gave birth within 2 months preceding the date of the interview as an indicator of woman's nutritional status. As per WHO recommendations, the woman was categorized as normal when BMI was 18.5–24.9 kg/m^2^, overweight if BMI was 25–29.9 kg/m^2^, and obese if BMI was ≥30 kg/m^2^.

The exposure variable of main interest was the frequency of watching television (TV), which was measured based on three categories (not watching television at all/watching television less than once a week/and watching television at least once a week).

Based on reviewing different literature, the following covariates were deemed relevant to the topic and for inclusion in the study such as the age of the respondents(15–24 years/25–39/40–49 years), residence (urban/rural), educational level (no education/primary/secondary/higher), marital status (never in union/currently in union/formerly in union), occupation (unemployed/nonagricultural/agricultural), wealth index (poorest/poorer/middle/richer/richest), alcohol drinking(yes/no), and parity (one birth/2 to 4 births/greater than 4 births).

### 2.4. Data Analysis

The data were analyzed using SPSS version 20. All statistical procedures incorporated complex sampling design analysis applied in the 2016 EDHS. Frequencies and weighted percentages of the variables were utilized to describe the profile of the study participants. Rao–Scott adjusted chi-square statistic was used to examine the distribution between the outcome variables (overweight and obesity) and each of the independent variables. Multivariate logistic regression analysis was conducted to assess the association between watching television with overweight and obesity among women by adjusting the covariates. Adjusted odds ratios (AOR) with 95% confidence interval were used to declare a statistically significant association.

## 3. Results

### 3.1. Participant's Characteristics

A total of 10,074 samples of reproductive age women were included and analyzed. About, 75.1% of respondents was rural residents, and 46.6% of the participants was found in the age between 25 and 39 years. Moreover, 46.8% of the respondents did not attend education. Only 17.5% of women watched television at least once per week. The prevalence of normal BMI, overweight, and obesity was 90.3%, 7.7%, and 2.0%, respectively (Table 1).

### 3.2. Factors Associated with Overweight and Obesity among Reproductive Age Woman in Ethiopia

All the variables were entered into multivariate logistic regression analysis. After adjusting for potential confounders by logistic regression, watching television at least once per week, age of the respondents (25–39 and 40–49 years), urban residence, attending higher education, and being rich in wealth index were positively associated with overweight and obesity. The study found that the odds of overweight and obesity was 1.79 (AOR: 1.79; 95% CI: 1.20–2.73) and 3.76 (AOR: 3.76; 95% CI: 2.04–6.95) times higher among women watching television at least once a week compared to women not watched at all, respectively.

In this study, the odds of overweight were higher among woman aged 25–39 years (AOR: 2.17; 95% CI: 1.25–3.77) and 40–49 years (AOR: 2.69; 95% CI: 1.45–5.00), urban residents (AOR: 1.76; 95% CI:1.17–2.6>5), attended higher education (AOR:2.11; 95% CI: 1.22–3.65), and richest in wealth index (AOR: 2.83; 95% CI:1.71–4.68). Likewise, the odds of obesity were higher among woman aged 25–39 years (AOR: 13.8; 95% CI: 5.47–34.9) and 40–49 years (AOR: 32.9; 95% CI: 12.1–89.7), attended higher education (AOR: 2.95; 95% CI: 1.25–6.97), and richest in wealth index (AOR: 4.23; 95% CI: 1.20–15.5) (Table 2).

## 4. Discussion

The study found that watching television at least once a week was significantly associated with both overweight and obesity among women of reproductive age in Ethiopia. The finding is coherent with studies conducted in the United States, Australia, Myanmar, Bangladesh, and India [18, 21–23, 29]. This may be due to watching television and displaces the physical activity, resulting in an overall decrease in energy expenditure [13].

The present study also showed that the odds of overweight were higher among women aged 25–39 years and 40–49 years, being urban residents, attending higher education, and richest in wealth index status. Likewise, the odds of obesity were also higher among woman aged 25–39 years and 40–49 years, attended higher education, and the richest in wealth index status.

The study has identified that the odds of overweight and obesity were higher among older women compared to younger women, which is consistent with findings from many other studies [30–33]. This may be explained by the intake of more energy-dense food as well as having less physical activity increases with age [34] and the association of higher age with changes in body composition (fat mass rises and also fat-free mass declines) [35, 36]. Overweight was associated with being urban residents compared to its counterparts. This finding is consistent with studies performed in Bangladesh, Myanmar, Ghana, and India [21, 22, 37–39]. This may be due to highly developed transportation, and technology results employment to be less labor-consuming, and higher coverage of electricity in urban areas in comparison to rural areas exposes the women to watch television frequently, by decreasing the physical activity.

The highest educational qualification has a strong positive association with women being overweight and obese in this study, which is also consistent with findings from other similar studies [30, 33, 40]. The expected reason for this might be higher educational levels which lead a woman into more sedentary occupations resulting in less physical activity.

Moreover, being the richest in wealth index status was associated with both overweight and obesity. This finding is supported by studies conducted in Bangladesh and India [30–32]. This could be explained by the consumption of energy-dense food products and the intake of higher fat increases with the rise in income [41].

### 4.1. Strength and Limitation of the Study

As it was conducted on a national sample of Ethiopian women, the findings would contribute a lot to interventions aimed at preventing overweight and obesity, which is the risk factor for different noncommunicable diseases. The findings of this study have important implications for policymakers and other concerned bodies. However, there are some limitations to consider. First, as cross-sectional data were used, we cannot assign causations to any of the associations between the identified factors and the outcomes of interest.

## 5. Conclusions

From the finding of the study, it is possible to demonstrate that watching television is associated with obesity among reproductive aged women in Ethiopia. Therefore, a social behavioral change communication campaign needs to be taken to improve awareness regarding the harmful consequences of watching television for long hours. Further research should be conducted among men and adolescents to determine whether this positive association exists among that target population as well.

## Figures and Tables

**Table 1:** Sociodemographic characteristics of the study participants and the prevalence of overweight and obesity across the independent variables, EDHS 2016 (*n* = 10,074).

Variables	Category	Total	Body mass index (BMI)			*p* value
			Normal	Overweight	Obese	
		*n* (wt.%)	*n* (wt.%)	*n* (wt.%)	*n* (wt.%)	
Age in years	15–24	3867 (37.7)	3555 (39.4)	278 (24.3)	34 (9.1)	<0.001^*∗*^
	25–39	4581 (46.6)	3745 (45.5)	628 (55.9)	208 (58.3)	
	40–49	1626 (15.7)	1234 (15.0)	280 (19.8)	112 (32.6)	

Residence	Urban	3903 (24.9)	2746 (20.7)	861 (59.6)	296 (82.8)	<0.001^*∗*^
	Rural	6171 (75.1)	5788 (79.3)	325 (40.4)	58 (17.2)	

Educational status	No education	4283 (46.8)	3863 (48.7)	334 (31.5)	86 (19.1)	<0.001^*∗*^
	Primary	3427 (34.6)	2922 (34.7)	400 (33.3)	105 (35.9)	
	Secondary	1531 (12.6)	1170 (11.8)	262 (20.0)	99 (22.4)	
	Higher	833 (5.9)	579 (4.8)	190 (15.3)	64 (22.6)	

Marital status	Never in union	2846 (26.9)	2518 (27.6)	278 (22.4)	50 (14.1)	<0.001^*∗*^
	Currently in union	6097 (63.2)	5136 (63.1)	717 (63.5)	244 (67.3)	
	Formerly in union	1131 (9.9)	880 (9.4)	191 (14.1)	60 (18.6)	

Occupation	Unemployed	4800 (47.4)	4163 (48.4)	486 (39.8)	151 (30.8)	<0.001^*∗*^
	Nonagricultural	3608 (31.8)	2784 (29.3)	634 (52.1)	190 (65.5)	
	Agricultural	1666 (20.8)	1587 (22.3)	66 (8.2)	13 (3.6)	

Wealth index	Poorest	1974 (15.0)	1854 (16.0)	104 (6.4)	16 (2.0)	<0.001^*∗*^
	Poorer	1250 (17.0)	1198 (18.2)	41 (6.9)	11 (3.5)	
	Middle	1284 (18.6)	1228 (20.0)	50 (7.9)	6 (0.4)	
	Richer	1386 (20.0)	1282 (20.9)	90 (12.7)	14 (7.7)	
	Richest	4180 (29.4)	2972 (25.0)	901 (66.2)	307 (86.3)	

Parity	1	1224 (16.8)	991 (16.7)	182 (19.2)	51 (14.3)	<0.001^*∗*^
	2–4	2840 (41.5)	2251 (40.3)	417 (48.2)	172 (64.4)	
	>4	2490 (41.6)	2190 (43.0)	226 (32.6)	74 (21.4)	

Drank alcohol	No	6470 (62.8)	5525 (63.2)	735 (60.5)	210 (56.6)	0.25
	Yes	3604 (37.2)	3009 (36.8)	451 (39.5)	144 (43.4)	

Frequency of watching television	Not at all	6038 (69.9)	5588 (73.6)	382 (40.7)	68 (17.5)	<0.001^*∗*^
	Less than once a week	1251 (12.6)	1029 (12.5)	173 (13.5)	49 (9.8)	
	At least once a week	2785 (17.5)	1917 (13.9)	631 (45.8)	237 (72.7)	

*n*: frequency; wt.%: weight percentage; ^*∗*^*p* value <0.05 is based on the adjusted *F*, which is a variant of the second-order Rao–Scott adjusted chi-square statistic.

**Table 2:** Multivariate analysis of the association between the frequency of watching television and overweight and obesity among reproductive age women in Ethiopia.

Variables	Category	Overweight		Obesity	
		COR (95% CI)	AOR (95% CI)	COR (95% CI)	AOR (95% CI)
Age in years	15–24	1	1	1	1
	25–39	1.99 (1.60–2.50)	2.17 (1.25–3.77)^*∗∗*^	5.57 (2.92–10.6)	13.8 (5.47–34.9)^*∗∗*^
	40–49	2.13 (1.64–2.78)	2.69 (1.45–5.00)^*∗∗*^	9.44 (5.47–16.3)	32.9 (12.1–89.7)^*∗∗*^

Residence	Urban	5.64 (4.30–7.40)	1.76 (1.17–2.65)^*∗∗*^	18.5 (10.1–33.6)	2.33 (0.75–7.18)
	Rural	1	1	1	1

Educational status	No education	1	1	1	1
	Primary	1.48 (1.19–1.85)	1.18 (0.86–1.63)	2.63 (1.51–4.58)	2.00 (0.95–4.21)
	Secondary	2.62 (1.96–3.50)	1.47 (0.89–2.43)	4.84 (2.85–8.21)	2.02 (0.95–4.28)
	Higher	4.95 (3.48–7.02)	2.11 (1.22–3.65)^*∗∗*^	12.1 (5.12–28.4)	2.95 (1.25-6.97)^*∗∗*^

Marital status	Never in union	1	1	1	1
	Married	1.24 (0.97–1.58)	1.52 (0.54–4.27)	2.08 (1.40–3.10)	0.56 (0.19–1.59)
	Formerly in union	1.85 (1.34–2.57)	1.79 (0.63–5.15)	3.88 (2.28–6.60)	0.72 (0.21–2.45)

Occupation	Unemployed	1	1	1	1
	Nonagricultural	2.16 (1.73–2.69)	1.06 (0.78–1.44)	3.50 (2.16–5.67)	1.18 (0.74–1.89)
	Agricultural	0.44 (0.29–0.67)	0.57 (0.37–0.88)	0.25 (0.08–0.74)	0.45 (0.14–1.43)

Wealth index	Poorest	1	1	1	1
	Poorer	0.96 (0.58–1.57)	0.79 (0.43–1.50)	1.55 (0.57–4.24)	1.84 (0.64–5.30)
	Middle	0.99 (0.61–1.62)	0.86 (0.49–1.52)	0.17 (0.06–0.53)	0.19 (0.06–0.63
	Richer	1.53 (0.99–2.37)	1.26 (0.73–2.17)	2.93 (1.07–8.06)	2.19 (0.76–6.40)
	Richest	6.69 (4.60–9.72)	2.83 (1.71-4.68)^*∗∗*^	27.6 (13.7–55.2)	4.23 (1.20-15.5)^*∗∗*^

Parity	1	1	1	1	1
	2–4	1.52 (1.09–2.11)	0.79 (0.48–1.32)	1.72 (0.87–3.35)	0.73 (0.29–1.81)
	>4	1.58 (1.20–2.07)	0.88 (0.63–1.24)	3.21 (2.14–4.82)	1.43 (0.89–2.26)

Drank alcohol	No	1	1	1	1
	Yes	1.12 (0.87–1.43)	0.99 (0.75–1.32)	1.31 (0.93–1.85)	0.90 (0.56–1.43)

Watching television	Not at all	1	1	1	1
	Less than once a week	1.94 (1.41–2.66)	1.04 (0.63–1.73)	3.28 (1.66–6.49)	1.76 (0.78–3.95)
	At least once a week	5.95 (4.68–7.56)	1.79 (1.20-2.73)^*∗∗*^	22.0 (12.8–37.6)	3.76 (2.04-6.95)^*∗∗*^

^*∗∗*^Significant association by AOR with 95% CI with a *p* value of less than 0.05.

## Data Availability

For this analysis, we used the USAID–DHS program 2016 Ethiopian demographic and health survey data set. To request the same or different data for another purpose, a new research project request should be submitted to the DHS program here: https://dhsprogram.com/data/Access-Instructions.cfm. The DHS program will normally review all data requests within 24–48 hours (during working days) and provide notification if access has been granted, or additional project information is needed before access can be granted. After receiving permission, the researcher can log in and select the specific data in the format they prefer.

## Abbreviations

AOR: Adjusted odds ratio
BMI: Body mass index
COR: Crude odds ratio
EDHS: Ethiopia demographic and health survey
SPSS: Statistical package for social science
TV: Television.
